# QTL mapping and candidate gene analysis of seed vigor-related traits during artificial aging in wheat (*Triticum aestivum*)

**DOI:** 10.1038/s41598-020-75778-z

**Published:** 2020-12-16

**Authors:** Huawei Shi, Wanghui Guan, Yugang Shi, Shuguang Wang, Hua Fan, Jinwen Yang, Weiguo Chen, Wenjun Zhang, Daizhen Sun, Ruilian Jing

**Affiliations:** 1 College of Agronomy, Shanxi Agricultural University, Taigu, 030801 People’s Republic of China; 2 Institute of Crop Science, Chinese Academy of Agricultural Sciences, Beijing, 100081 People’s Republic of China

**Keywords:** Genetics, Molecular biology

## Abstract

High vigor seeds have greater yield potential than those with low vigor; however, long-term storage leads to a decline in this trait. The objective of this study was to identify quantitative trait loci (QTLs) for seed vigor-related traits under artificial aging conditions using a high-density genetic linkage map of wheat (*Triticum aestivum*) and mine the related candidate genes. A doubled haploid population, derived from a cross between Hanxuan 10 × Lumai 14, was used as the experimental material. Six controlled-environment treatments were set up, i.e. the seeds were aged for 0, 24, 36, 48, 60, and 72 h at a high temperature (48 °C) and under high humidity (relative humidity 100%). Eight traits including seed germination percentage, germination energy, germination index, seedling length, root length, seedling weight, vigor index, and simple vigor index were measured. With the prolongation of artificial aging treatment, these traits showed a continuous downward trend and significant correlations were observed between most of them. A total of 49 additive QTLs for seed vigor-related traits were mapped onto 12 chromosomes (1B, 2D, 3A, 3B, 3D, 4A, 4D, 5A, 5B, 5D, 6D, and 7A); and each one accounted for 6.01–17.18% of the phenotypic variations. Twenty-five pairs of epistatic QTLs were detected on all chromosomes, except for 5D, 6A, and 7D, and each epistasis accounted for 7.35–26.06% of the phenotypic variations. Three additive QTL hot spots were found on chromosomes 5A, 5B, and 5D, respectively. 13 QTLs, *QGEe5B*, *QGIe5B*, *QSLc5B*, *QSLd5B*, *QSLf5B*, *QRLd5B*, *QRLe5B*, *QRLf5B*, *QVId5B*, *QVIe5B*, *QVIf5B*, *QSVId5B*, and *QSVIe5B*, were located in the marker interval AX-94643729 ~ AX-110529646 on 5B and the physical interval 707,412,449–710,959,479 bp. Genes including *TRAESCS5B01G564900, TRAESCS5B01G564200, TRAESCS5B01G562600, TraesCS5B02G562700, TRAESCS5B01G561300, TRAESCS5B01G561400*, and *TRAESCS5B01G562100*, located in this marker interval, were found to be involved in regulating the processes of carbohydrate and lipid metabolism, transcription, and cell division during the germination of aging seeds, thus they were viewed as candidate genes for seed viability-related traits. These findings provide the basis for the seed-based cloning and functional identification of related candidate genes for seed vigor.

## Introduction

Seeds not only contain all of a plant’s genetic information, but are also important agricultural production materials. Seed vigor is the primary index used to measure seed quality and is also an important factor in determining yield^1^. Seeds with high vigor demonstrate a high percentage of germination, uniform seedlings that grow quickly, enhanced resistance to stress, and greater productivity. However, the percentage of germination in seeds with low vigor is small and seedling growth is weak^2^. Therefore, it is necessary to investigate seed vigor-related traits, explore their genetic laws, and combine modern breeding methods to select varieties with high-vigor in order to promote the development of agricultural production^3–5^.

Seed aging refers to the natural decline of seed vigor. The temperature and humidity during seed storage greatly impact seed vigor and the aging process of seeds is often accelerated under conditions of high temperature and high humidity^6^. As it would take a long time to study the effect of storage on vigor under normal conditions, aging can be accelerated artificially, in order to speed up the process of investigation. Accelerated aging is a method recommended by the International Seed Testing Association (ISTA)^7^.

The peroxidation of membrane lipids and proliferation of radicals are two important reasons for decreases in seed vigor^8^. Lipoxygenase (LOX) is one of the key enzymes that catalyze the metabolism of unsaturated fatty acids. Following an increase in LOX activity, levels of unsaturated fatty acids decrease, fatty acids are oxidized, free radicals form, then seeds deteriorate quickly^9^. Also, when seeds are stimulated by an adverse external environment or undergo the natural aging process, a series of physiological and biochemical changes occurs within, which leads to protein denaturation, damage to the nucleic acid synthesis system, or injury to the cell membrane, resulting in seed deterioration and decreased vigor^10^.

The development of functional genomics has promoted the study of seed vigor. In *Arabidopsis*, overexpression of *ZmGOLS2* and *ZmRS*, or overexpression of *ZmGOLS2* alone, can significantly increase the oligosaccharide content and enhance seed vigor^11^. Cheng et al.^12^ found that lectin-like receptor protein kinase (LecRK) contributed to seed germination and innate immunity in rice and demonstrated that a knockout of *OslecRK* inhibiteds the expression of the α-amylase gene, thus reducing seed vigor^12^. Additional factors related to seed vigor are small heat shock proteins (sHSPs). Kaur et al.^13^ found that the cytoplasmic sHSP oshsp18.2 can improve seed vigor in rice by reducing the harmful accumulation of active oxygen in seeds. In addition, Châtelain et al.^14^ found that methionine sulfoxide re ductase (MSR) can increase seed life by regulating oxidative repair in *Arabidopsis* seeds. With acceleration in aging, proteins containing isoaspartic acid over-accumulate in embryos. Protein repair L-isoaspartyl methyltransferase1 (Ospimt1) can improve seed vigor by repairing the harmful proteins containing isoaspartic acid^15,16^.

Seed vigor is a complex agronomic trait^9^. It has many measures including average germination rate (AGR), germination index (GI), vigor index (VI)^17^, seed root length (SRL), seedling length (SL), seed wet weight (SWW), and seed dry weight (SDW), etc^18^. These traits are all quantitative and controlled by multiple genes. There have been many reports on quantitative trait loci (QTL) analyses of seed vigor traits over recent years in *Arabidopsis*, rice, wheat, rape, maize, and barley, etc^19–23^. Progress has also been made in the fine mapping of seed vigor QTLs. Xie et al.^24^ mapped eight seed vigor-related QTLs using a recombinant inbred line population of an indica and japonica rice hybrid and shortened the regions of two main QTLs, *qsv1* and *qsv5c* of rice chromosomes 1 and 5, to physical intervals of 1.13 Mb and 400 kb, respectively. Li et al.^25^ detected 19 QTLs related to seed vigor on 12 chromosomes of rice and located *qgp9* in a region of 92.6 kb between two sequenced tagged site (STS) markers on chromosome 9 of rice. Abe et al.^26^ located a QTL controlling seedling height at the end of the long arm of rice chromosome 3 and predicted that the candidate gene was a GA20 oxidase gene (*OsGA20ox1*).

In recent years, most studies on wheat seed vigor have focused on revealing the physiological mechanisms during artificial aging, following seed priming, and after other treatments, such as the changes of antioxidant system, storage substances, endogenous hormones and harmful substances, while the relevant molecular mechanism has rarely been discussed. In this study, a doubled haploid (DH) population of wheat (*Triticum aestivum*) was artificially aged and eight seed vigor-related traits were determined. The changing trend of each trait after aging treatment was analyzed and correlation analysis was performed. A high-density linkage genetic map was used for QTL mapping of related traits and the candidate genes were screened out. These candidate genes will provide the basis for breeding and improvement of new wheat varieties with high vigor seeds.

## Results

### Phenotypic changes to seed vigor-related traits in a wheat DH population under different aging conditions

In the current study, eight seed vigor indexes of a wheat DH population were measured under artificial aging conditions. As the period of aging treatment increased, the eight seed vigor-related traits in the DH population showed a gradually decreasing trend (Supplementary Table S1). At 0 h of aging treatment, the VI, SVI, SL, RL, and SW values for the female parent Hanxuan 10 were significantly higher than those of the male parent Lumai 14. At 24 and 36 h after the onset of aging treatment, the other seven traits in Hanxuan 10, except RL, were significantly greater than those of Lumai 14. After 48 h of aging treatment, the VI, SVI, SL, and SW values for Hanxuan 10 were significantly or extremely significantly greater than those for Lumai 14. After 60 h of aging treatment, the GP and GE values for Hanxuan 10 were significantly greater than those for Lumai 14. After 72 h of aging treatment, the GP, GI, SW, VI, and SVI values for Hanxuan 10 were significantly higher than those for Lumai 14.

**Figure 1:**
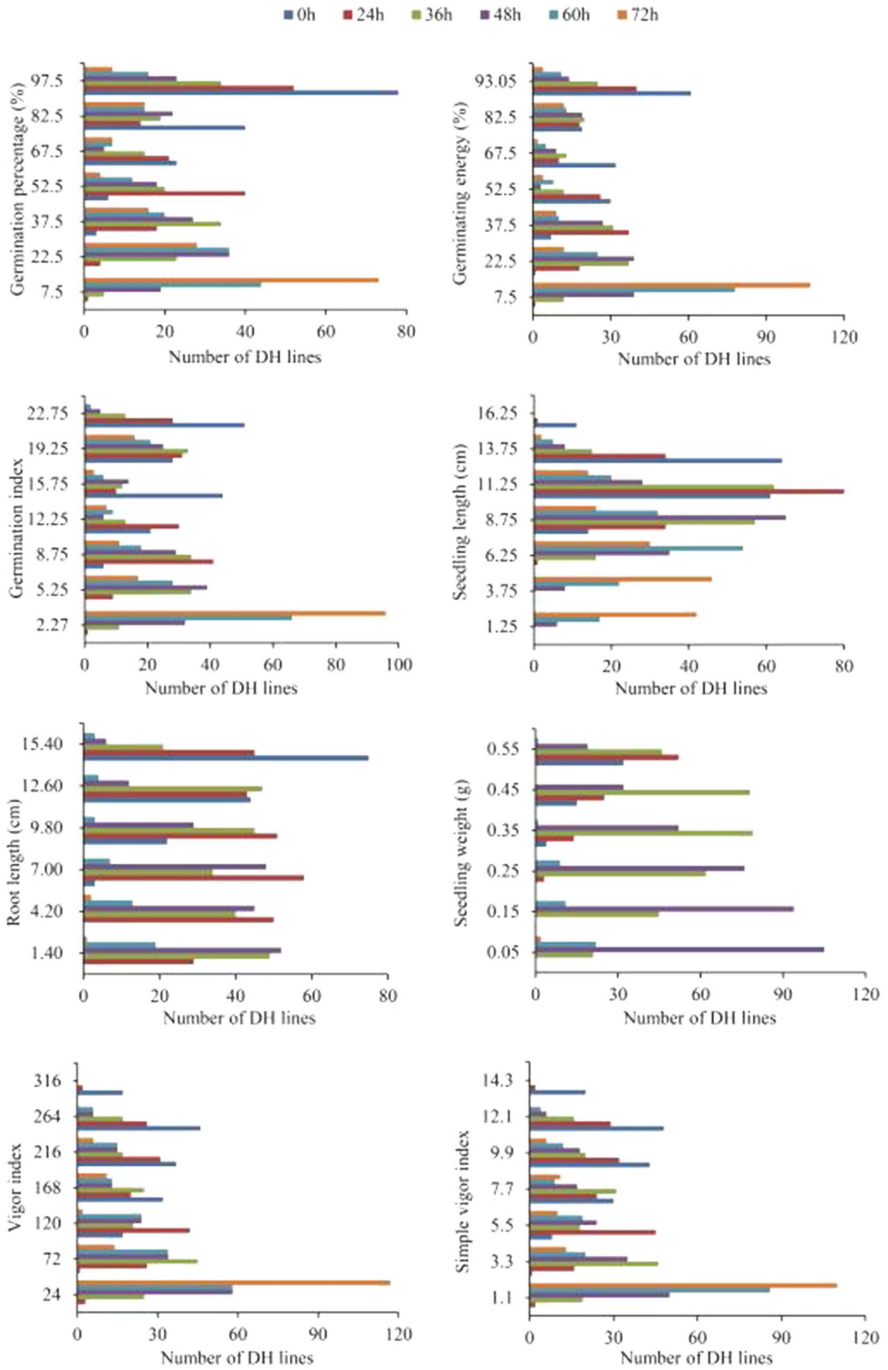
Frequency distribution of seed vigor-related traits in wheat DH population.

Under the six treatment conditions, eight seed vigor-related traits in the DH population exhibited the phenomenon of superaffinity separation, indicating that the genes controlling each trait were widely separated within the population. The variation coefficient for each trait in the population was more than 14% and the absolute values of skewness and kurtosis coefficients for most traits were less than 1, which conformed to normal distribution (Supplementary Table S1). The absolute values of skewness coefficient and kurtosis coefficient of some characters, such as VIf and SVIf, are greater than one, indicating that there are major QTLs. Under aging treatment, the eight seed vigor-related traits of the DH population were continuously distributed (Fig. 1), indicating that each trait was controlled by multiple genes. The heritability of each seed vigor-related trait was above 81% and the heritability of a large portion of traits reached more than 95%, indicating that these eight traits were strongly affected by genetic factors (Supplementary Table S1).

### Correlation of seed vigor traits in the DH population

**Figure 2:**
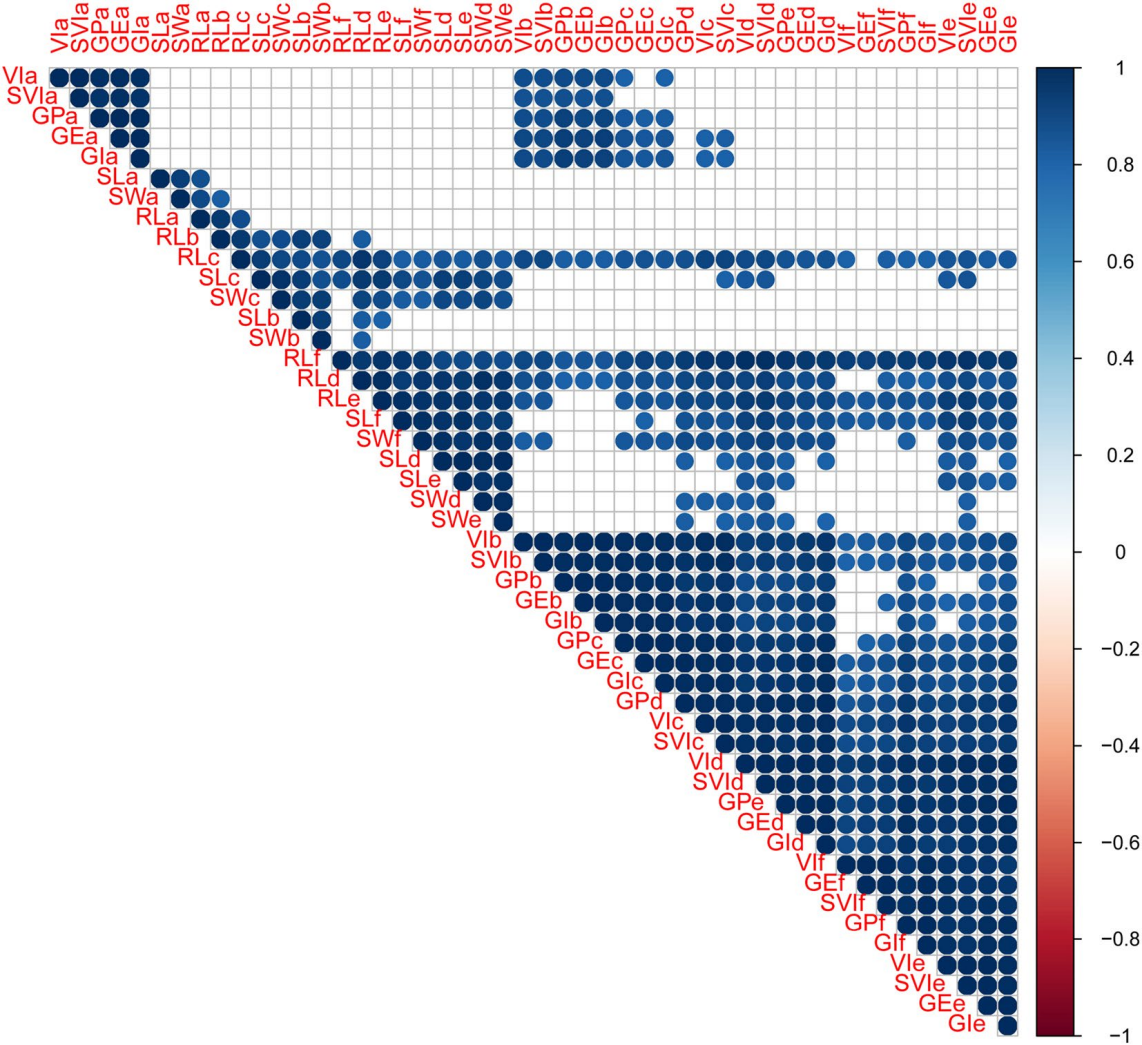
Correlation of seed vigor-related traits in wheat DH population.

Under the same treatment, the GP, GE, GI, VI and SVI values were significantly or extremely correlated in the DH population, while SW, SL, and RL were significantly or extremely correlated. Under different aging conditions, most of the seed vigor-related traits were significantly or extremely significantly correlated (Fig. 2).

**Figure 3:**
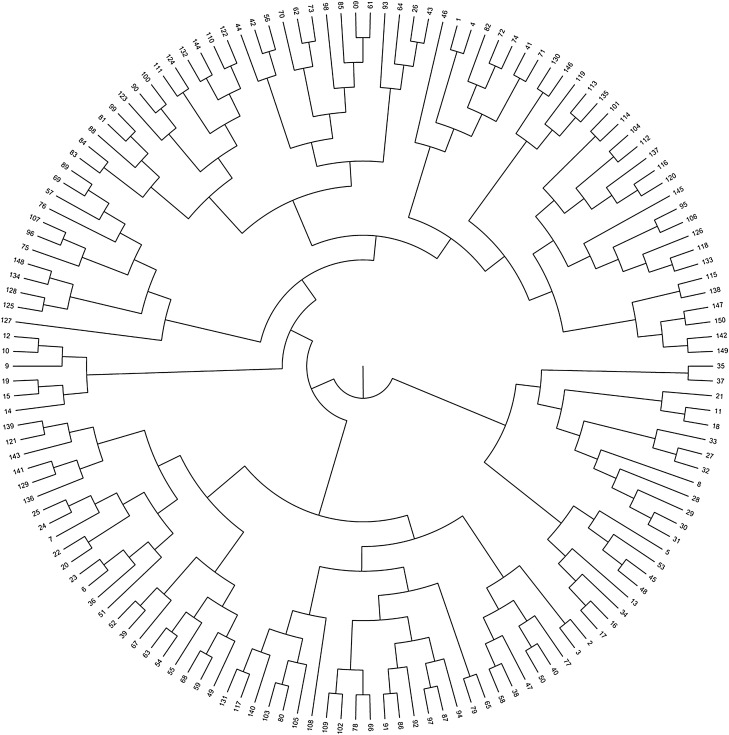
Systematic clustering of wheat DH population.

Systematic clustering was conducted using the 150 lines of the DH population, were divided into nine types (Fig. 3).

### Additive QTLs for seed vigor-related traits in the DH population of wheat

QTL mapping was performed on eight vigor-related traits of the DH population under six different treatment conditions. A total of 49 additive QTLs for wheat seed vigor-related traits were detected and each ones contributed 6.01–17.18% of the phenotypic variation. The logarithm of odds scores (LOD score) ranged from 3.54 to 7.28, distributed on 12 chromosomes including 1B, 2D, 3A, 3B, 3D, 4A, 4D, 5A, 5B, 5D, 6D, and 7A, respectively. Among them, 43 elitealleles of additive QTLs were from the female parent Hanxuan 10 and six were from the male parent Lumai 14. The chromosome with the most QTL loci was 5D, which contained a total of 18 additive QTLs, followed by chromosome 5B, with 14 additive QTLs. Additionally, chromosome 5A contained 7 additive QTLs and chromosome 2D contained 2 additive QTLs. One additive QTL locus each was detected on chromosomes 1B, 3A, 3B, 3D, 4A, 4D, 6D, and 7A, respectively (Table 1).

**Table 1:** Additive QTLs for seed vigor traits in wheat DH lines using the ICIM method.

Trait	QTL	Chr	Position (cM)	Left marker	Left CI	Right marker	Right CI	LOD score	PVE (%)	Add
GPe	*QGPe5D*	5D	19	Xgdm3	18.5	AX-109095227	19.5	5.02	11.23	0.1133
GPf	*QGPf5D*	5D	19	Xgdm3	18.5	AX-109095227	20.5	6.47	14.65	0.1240
GEd	*QGEd5D*	5D	19	Xgdm3	18.5	AX-109095227	20.5	5.14	11.60	0.1130
GEe	*QGEe5B*	5B	229	AX-94643729	226.5	AX-110529646	229	4.53	11.77	0.0989
GEe	*QGEe5D*	5D	19	Xgdm3	18.5	AX-109095227	19.5	5.73	14.99	0.1120
GEf	*QGEf5A-1*	5A	19	AX-111662464	12.5	AX-95683796	20.5	3.55	7.07	0.0778
GEf	*QGEf5A-2*	5A	15	AX-94524640	12.5	AX-89452330	21.5	4.29	8.64	− 0.0859
GEf	*QGEf5D*	5D	13	AX-89752452	11.5	AX-110129540	13.5	5.05	10.39	0.0949
GId	*QGId5D*	5D	19	Xgdm3	18.5	AX-109095227	20.5	4.63	10.55	2.3675
GIe	*QGIe5B*	5B	229	AX-94643729	226.5	AX-110529646	229	3.94	9.77	2.0943
GIe	*QGIe5D*	5D	19	Xgdm3	18.5	AX-109095227	19.5	6.11	15.49	2.6455
GIf	*QGIf5D*	5D	19	Xgdm3	18.5	AX-109095227	20.5	6.41	14.34	2.5290
SLa	*QSLa4D*	4D	16	AX-89703298	14.5	AX-89421921	18.5	3.54	8.31	0.5019
SLa	*QSLa5A*	5A	68	AX-95148334	65.5	Xgwm156	69.5	4.45	11.10	0.5892
SLb	*QSLb5A*	5A	69	Xgwm156	68.5	Xgwm415	71.5	5.76	17.18	0.6678
SLc	*QSLc5A*	5A	69	Xgwm156	68.5	Xgwm415	72.5	4.24	11.12	0.6168
SLc	*QSLc5B*	5B	226	AX-94643729	215.5	AX-110529646	229	4.69	12.27	0.6395
SLd	*QSLd3A*	3A	1	AX-110122723	0	AX-89691263	1.5	3.64	6.01	− 0.7172
SLd	*QSLd3B*	3B	48	Xwmc231	47.5	Xwmc231	48.5	6.17	10.68	− 0.9396
SLd	*QSLd5A*	5A	51	Xgwm205.1	50.5	AX-95685047	51.5	3.99	7.49	0.7803
SLd	*QSLd5B*	5B	229	AX-94643729	224.5	AX-110529646	229	4.17	7.03	0.7527
SLe	*QSLe1B*	1B	118	Xwmc44	115.5	AX-108745931	119.5	3.65	8.08	− 0.9160
SLe	*QSLe5A*	5A	69	Xgwm156	68.5	Xgwm415	71.5	7.28	17.14	1.3485
SLf	*QSLf5B*	5B	229	AX-94643729	224.5	AX-110529646	229	3.68	10.13	1.0635
SLf	*QSLf5D*	5D	19	Xgdm3	18.5	AX-109095227	19.5	4.60	12.85	1.2015
RLd	*QRLd2D*	2D	162	AX-108820505	160.5	AX-109351504	164.5	5.71	12.19	1.0691
RLd	*QRLd5B*	5B	229	AX-94643729	225.5	AX-110529646	229	3.69	7.76	0.8432
RLe	*QRLe2D*	2D	162	AX-108820505	160.5	AX-109351504	164.5	4.99	10.77	1.0277
RLe	*QRLe5B*	5B	229	AX-94643729	225.5	AX-110529646	229	4.25	9.24	0.9411
RLf	*QRLf5B*	5B	229	AX-94643729	225.5	AX-110529646	229	4.81	11.09	0.9670
RLf	*QRLf5D*	5D	19	Xgdm3	18.5	AX-109095227	20.5	4.30	9.79	0.9109
SWa	*QSWa3D*	3D	25	AX-94550177	42.5	AX-111713694	49.5	5.07	10.01	− 0.0178
SWc	*QSWc4A*	4A	91	AX-94581303	90.5	Xwmc468	91.5	5.77	15.58	− 0.0201
SWe	*QSWe7A*	7A	143	AX-94502577	140.5	Xgwm282	145.5	3.64	10.29	0.0269
VIc	*QVIc5D*	5D	18	AX-110091432	15.5	Xgdm68	18.5	4.34	10.38	26.2836
VId	*QVId5B*	5B	229	AX-94643729	225.5	AX-110529646	229	4.19	10.25	24.1877
VId	*QVId5D*	5D	19	Xgdm3	18.5	AX-109095227	19.5	5.66	14.01	28.3611
VIe	*QVIe5B*	5B	229	AX-94643729	226.5	AX-110529646	229	4.79	11.46	24.2392
VIe	*QVIe5D*	5D	19	Xgdm3	18.5	AX-109095227	19.5	6.95	17.06	29.6617
VIf	*QVIf5B*	5B	229	AX-94643729	225.5	AX-110529646	229	4.09	9.61	19.3606
VIf	*QVIf5D*	5D	19	Xgdm3	18.5	AX-109095227	19.5	6.28	15.13	24.3613
SVIa	*QSVIa6D*	6D	25	AX-111602987	23.5	AX-89689923	26.5	4.17	10.13	0.7989
SVIc	*QSVIc5D*	5D	18	AX-110091432	16.5	Xgdm68	18.5	4.11	9.86	1.1407
SVId	*QSVId5B*	5B	229	AX-94643729	225.5	AX-110529646	229	3.67	9.26	1.0514
SVId	*QSVId5D*	5D	19	Xgdm3	18.5	AX-109095227	19.5	4.99	12.72	1.2360
SVIe	*QSVIe5B*	5B	229	AX-94643729	225.5	AX-110529646	229	4.49	11.09	1.0948
SVIe	*QSVIe5D*	5D	19	Xgdm3	18.5	AX-109095227	19.5	6.31	15.93	1.3158
SVIf	*QSVIf5B*	5B	229	AX-94643729	225.5	AX-110529646	229	4.25	9.87	0.9306
SVIf	*QSVIf5D*	5D	19	Xgdm3	18.5	AX-109095227	19.5	6.73	16.14	1.1929

GP, GE, GI, SL, RL, SW, VI, SVI = Germination Percentage, Germinating Energy, Germination Index, Seedling Lengh, Root Length, Seedling Weight, Vigor Index, and Simple Vigor Index, respectively. PVE = percentage of phenotypic variance explained by the QTL, Add = additive effect. a, b, c, d, e, and f represent aging for 0 h, 24 h, 36 h, 48 h, 60 h and 72 h, respectively.

### Epistatic QTLs

A total of 25 pairs of epistatic QTLs were detected, which were distributed on all of the wheat chromosomes, except 5D, 6A, and 7D. Their LOD Scores ranged from 5.03 to 9.14, their contribution to phenotypic variation were 7.35–26.06%, and epistatic interaction effects ranged from 0.02 to 61.23 (Table 2). Among them, there were 14 pairs of epistatic QTLs with a negative interaction effects, indicating that the parent epistasis effect was smaller than the recombinant epistasis effect. Eleven pairs of epistatic QTLs had positive interaction effect values, indicating that the recombinant epistasis effects were smaller than the parent epistasis effect.

**Table 2:** Epistatic QTLs for seed vigor traits in wheat DH population.

Trait	QTL 1	Chr	Position 1 (cM)	Flanking marker 1	QTL 2	Chr	Position 2 (cM)	Flanking marker 2	LOD	PVE (%)	AA
GPe	*QGPe6B*	6B	70	Xwmc269.3–Xwmc494	*QGPe7B*	7B	50	AX-108946649–AX-108772866	5.22	13.21	− 0.13
GPf	*QGPf1D*	1D	15	Xcwm170-AX–109860229	*QGPf3D*	3D	70	AX-110558400–AX-110394178	5.64	15.34	− 0.11
GEb	*QGEb3A*	3A	35	AX-110673267–AX-109444631	*QGEb6D*	6D	0	AX-94474462–AX-108941325	5.16	16.08	− 0.10
GEe	*QGEe2A*	2A	20	Xcwm138.2–AX-110686688	*QGEe3B*	3B	0	AX-111125857–AX-111663217	5.18	14.68	− 0.10
GEf	*QGEf3B*	3B	0	AX-111125857–AX-111663217	*QGEf6B*	6B	105	AX-110928656–AX-109883174	6.07	17.68	0.10
GIf	*QGIf1D*	1D	15	Xcwm170–AX-109860229	*QGIf3D*	3D	75	AX-108859112–AX-108735265	5.03	12.35	− 2.14
GIf	*QGIf3B*	3B	0	AX-111125857–AX-111663217	*QGIf6B*	6B	105	AX-110928656–AX-109883174	5.60	14.02	2.24
SLa	*QSLa1D*	1D	145	AX-111581326–AX-111644713	*QSLa7B*	7B	65	AX-110380079–Xwmc269.1	5.15	12.46	− 0.61
RLb	*QRLb5B*	5B	150	AX-110671305–AX-111586069	*QRLb6B*	6B	0	Xcwm449–AX-109378013	5.04	14.20	1.02
RLe	*QRLe1B*	1B	50	Xgwm582–AX-95230830	*QRLe3B*	3B	145	AX-108729033–AX-110061327	5.28	22.61	− 1.04
SWa	*QSWa1A*	1A	40	Xwmc336–AX-89414766	*QSWa3A*	3A	10	EST47–AX-95661215	5.38	15.22	− 0.02
SWa	*QSWa1B*	1B	85	AX-94867126–AX-111073230	*QSWa5A*	5A	0	AX-94515371–AX-109485082	5.60	12.75	− 0.02
SWa	*QSWa7A*	7A	0	Xcwm461.2–Xcwm462.2	*QSWa7A*	7A	75	AX-94712676–AX-110011761	5.30	12.79	0.02
SWb	*QSWb3D*	3D	260	AX-110945080–AX-89662133	*QSWb4A*	4A	145	AX-95143704–AX-94460900	6.50	18.57	− 0.02
SWc	*QSWc3D*	3D	260	AX-110945080–AX-89662133	*QSWc4A*	4A	150	AX-94460900–AX-9525105	5.17	15.50	− 0.02
SWd	*QSWd1B*	1B	25	AX-89662088–EST122	*QSWd1B*	1B	100	AX-111496745–AX-108753515	5.84	14.91	0.03
SWd	*QSWd2D*	2D	25	AX-109783007–Xgwm296.1	*QSWd4A*	4A	20	AX-110532752–AX-111118108	5.05	11.90	0.02
SWe	*QSWe1B*	1B	30	EST122–Xcwm548	*QSWe1B*	1B	125	AX-111708758–AX-94912518	5.29	19.33	0.03
VIb	*QVIb3A*	3A	125	AX-110161651–AX-111635376	*QVIb4D*	4D	25	AX-110564616–AX-111354775	5.12	14.06	27.12
VIb	*QVIb3A*	3A	35	AX-110673267–AX-109444631	*QVIb6D*	6D	0	AX-94474462–AX-108941325	5.49	13.60	− 27.98
VIe	*QVIe5B*	5B	115	AX-110167647–AX-95232642	*QVIe6B*	6B	95	AX-111604989–AX-11098608	5.09	20.60	24.43
VIe	*QVIe2B*	2B	20	Xwmc317–AX-111601433	*QVIe2B*	2B	30	AX-109283083–AX-95101397	9.14	26.06	− 61.23
VIf	*QVIf3B*	3B	0	AX-111125857–AX-111663217	*QVIf6B*	6B	105	AX-110928656–AX-109883174	5.39	7.35	21.35
SVIf	*QSVIf2B*	2B	20	Xwmc317–AX-111601433	*QSVIf2B*	2B	25	AX-108874208–AX-110043446	7.24	23.65	− 2.72
SVIf	*QSVIf3B*	3B	0	AX-111125857–AX-111663217	*QSVIf6B*	6B	105	AX-110928656–AX-109883174	5.41	7.37	1.01

Positive value for epistatic effect means the greater genetic values of parental types than those of recombination types, while the negative value indicates the opposite scenarios. PVE = percentage of phenotypic variance explained by each QTL, AA = Additive-by-additive interaction effect.

### Additive QTL hotspots and candidate genes

**Figure 4:**
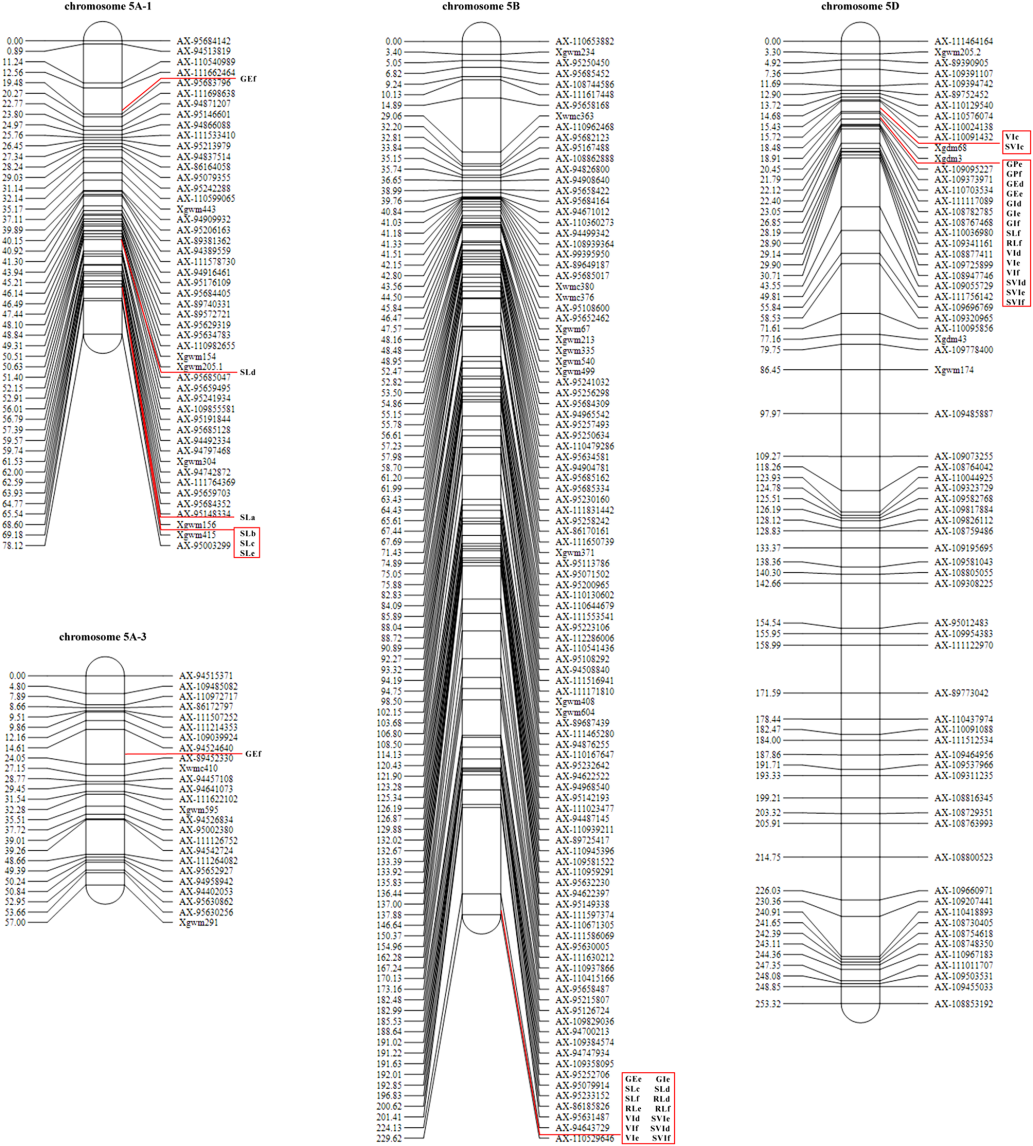
Distribution of additive QTL hotspot regions in seed vigor traits of wheat DH population.

Using the ICIM methods, three additive QTL hotspots were found. The first one was within the marker interval Xgwm156 ~ Xgwm415 on chr 5A, which contains three QTLs related to SL under different aging treatments. The second one was within marker interval Xgdm3 ~ AX-109095227 on chr 5D, which contains fifteen QTLs related to GE, GI, SL, RL, VI, and SVI under different aging treatments. The last one was within marker interval AX-94643729 ~ AX-110529646 on chr 5B, it contains thirteen QTLs related to GE, GI, SL, RL, VI, and SVI under different aging treatments (Fig. 4).

By the GCIM methods, three QTLs related to SL under different aging treatments were detected on the marker Xgwm415 on chr 5A, two QTLs related to SL and VI under different aging treatments were detected on the marker Xgdm3 on chr 5D, and seven QTLs related to SL and RL under different aging treatments were detected on the marker AX-110529646 on chr 5B (Supplementary Table S2). Clearly, the QTL hotspots found by the two methods are consistent.

The physical distances of the first and second hotspots are too long and the number of genes is too large, so it is difficult to predict candidate genes. For the last one, the physical distance between the two markers AX-94643729 and AX-110529646 is 707412449-710959479 bp.

According to the Chinese Wheat Complete Genome Reference Sequence (*IWGSC RefSeqv1.0*) published by the International Wheat Whole Genome Sequencing Consortium, a total of 56 genes were found in this marker range and gene annotation was carried out by referring to the website https://plants.ensembl.org/index.html. It was found that seven genes, *TRAESCS5B01G564900, TRAESCS5B01G564200, TRAESCS5B01G562600, TraesCS5B02G562700, TRAESCS5B01G561300, TRAESCS5B01G561400*, and *TRAESCS5B01G562100* may be related to seed vigor (Table 3).

**Table 3:** Genetic information of additive QTL hotspots in wheat DH population.

Gene ID	Gene annotation
*TraesCS5B01G564900*	Invertase/pectin methylesterase inhibitor family protein
*TraesCS5B01G561300*	Cytokininriboside 5′-monophosphate phosphoribohydrolase
*TraesCS5B01G561400*	Cytokininriboside 5′-monophosphate phosphoribohydrolase
*TraesCS5B01G562100*	WD40 repeat-containing protein
*TraesCS5B01G562600*	GDSL esterase/lipase
*TraesCS5B01G562700*	GDSL esterase/lipase
*TraesCS5B01G564200*	Trihelix transcription factor

### GO and KEGG analyses of candidate genes

**Figure 5:**
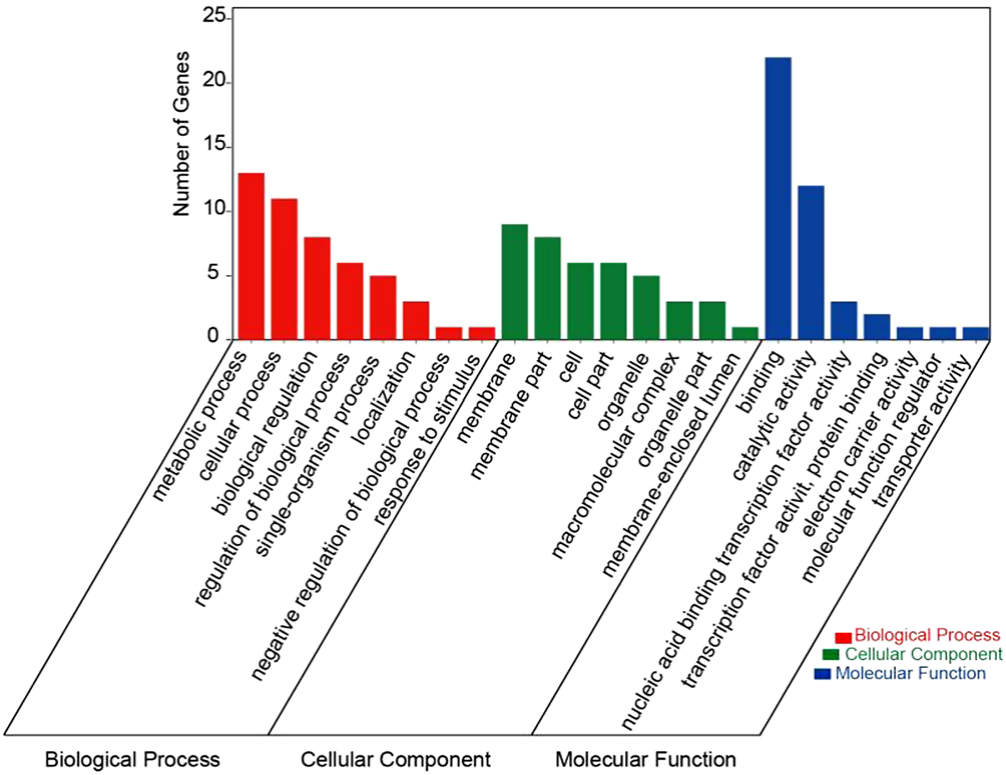
GO annotation clustering map of the candidate genes.

We performed GO (gene ontology) enrichment analysis on candidate genes (Supplementary Table S3, Fig. 5). The genes *TraesCS5B02G561300* and *TraesCS5B02G561400* appeared in four terms, and the response pathways included metabolic process, catalytic activity, cellular process, and biological regulation. The gene *TraesCS5B02G562100* appeared in three terms, and the response pathways involved include binding activity and membrane part. Gene *TraesCS5B02G564900* appeared in two terms, the response pathways included metabolic process and biological regulation. The genes *TraesCS5B02G562600*, *TraesCS5B02G562700* appeared in one term, and the participating response pathway was catalytic activity.

**Figure 6:**
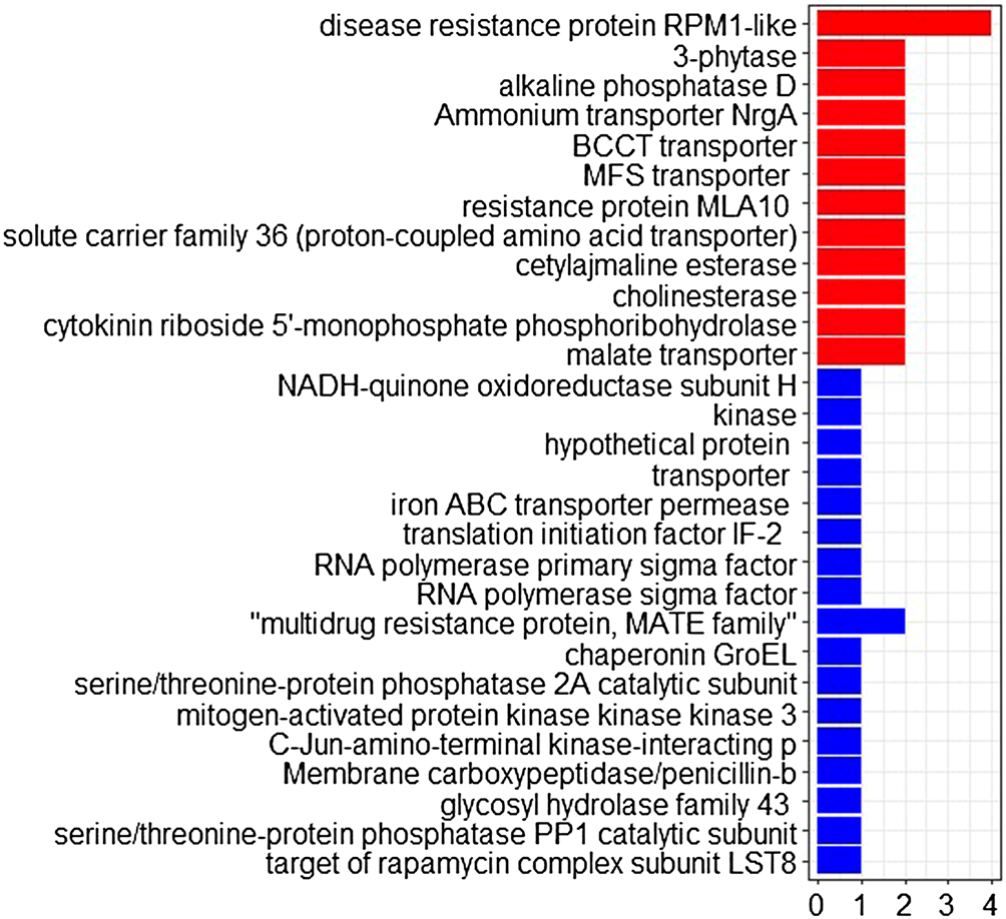
Pathway distribution map of the candidate genes.

KEGG (Kyoto Encyclopedia of Genes and Genomes) analysis was performed to mine candidate genes (Supplementary Table S4, Fig. 6). Genes *TraesCS5B02G562600* and *TraesCS5B02G562700* were significantly enriched in five metabolic pathways of 3-phytase, alkaline phosphatase D, solute carrier family 3, cetylajmaline esterase, and cholinesterase. The gene *TraesCS5B02G562100* is significantly enriched in eight metabolic pathways, which include nucleotides, protein metabolism and ribosome assembly, etc. The gene *TraesCS5B02G564200* was significantly enriched in the three metabolic pathways of C-Jun-amino-terminal kinase-interacting p, serine and arginine repetitive matrix 1, and ATP-dependent (S)-NAD(P)H-hydrate dehydra. The genes *TraesCS5B02G561300* and *TraesCS5B02G561400* were significantly enriched in the three metabolic pathways of cytokinin riboside, 5′-monophosphate, and phosphoribohydrolase.

## Discussion

### Significant phenotypic correlation among wheat seed vigor-related traits

Seed vigor is affected by various external conditions. Under high temperature and humidity, a large number of seeds at different degrees of aging can be obtained, which is conducive to further research on vigor theory and applications. This method has provided useful results for investigations into the seed vigor of various crops. Ye^27^ studied the response to high temperatures of rice seeds containing different water contents. Under long-term high temperature conditions, seed vigor and the longevity of high water content seeds were low. Han et al.^2^ analyzed the GP and Ge of maize seeds under artificial aging and carried out relevant QTL mapping.

In this study, the GP, GE, GI, SL, RL, SW, VI, and SVI values of the DH population decreased continuously with the increasing period of aging treatment. There were significant differences in seed vigor-related traits between parents Hanxuan 3 and Lumai 14 of DH population under artificial aging treatment, which may be due to the great differences in the source and physiological characteristics of parents. The absolute values of skewness and kurtosis coefficient of each trait in DH population are mostly less than one, which conforms to normal distribution. The absolute values of skewness coefficient and kurtosis coefficient of some traits, such as VIf and SVIf, are greater than one, indicating that there are major QTLs. Under different aging conditions, GP, GE, GI, VI, and SVI were significantly positively correlated, while SW, SL, and RL were also significantly correlated under most aging conditions. Under different aging conditions, GP, GE, GI, VI, and SVI were significantly positively correlated, while SW, SL, and RL were also significantly correlated under most aging conditions. QTL mapping was carried out for the eight seed vigor-related traits and four additional QTL hot spots were found. First, three QTLs related to SL under different aging treatments was detected in the marker range xgwm156 ~ xgwm415 on chromosome 5A. Second, QTLs related to GE, GI, SL, RL, VI, and SVI were detected in the marker interval Xgdm3 ~ AX-109095227 on chromosome 5D under different aging treatments. Third, a QTL associated with VI and SVI was detected on chromosome 5D in marker interval AX-110091432 ~ Xgdm68. Fourth, a QTL associated with six traits including GE, GI, SL, RL, VI, and SVI was detected on chromosome 5B under different aging treatments in the marker interval AX-94643729 ~ AX-110529646. Further mapping showed that these QTLs may be due to multiple effects or close linkage, which provided good molecular genetic evidence for the significant phenotypic correlation among the traits.

### New QTLs for wheat seed vigor-related traits

In recent years, great progress has been made in the QTL mapping of various important traits including seed vigor in many crops. So far, research has focused on a limited number of plants such as rice, *Arabidopsis*, cabbage, barley, and sorghum. For example, Shi et al.^28^ used two maize recombinant inbred line populations under low temperatures to detect 26 QTLs related to seed vigor such as GP and GI. These QTLs were located on all chromosomes, except chromosome 10. However, there are few studies on QTL mapping of wheat seed vigor. Misheva et al.^29^ performed QTL mapping on GP and SL of aged wheat seeds under osmotic stress. The results showed that 20 QTLs were detected and distributed on chromosomes 1D, 2D, 4D, 5D, and 7D. Arif et al.^23^ detected loci associated with seed longevity in bread wheat on chromosomes 1D, 2A, 7B, and 7D. Zuo et al.^30^ found that chromosomes 2D, 3D, 4A, and 6B of wheat are important related to six seed germination parameters. In this study, 49 additive QTLs of wheat seed vigor-related traits were detected and found to be distributed on 12 chromosomes including 1B, 2D, 3A, 3B, 3D, 4A, 4D, 5A, 5B, 5D, 6D, and 7A. Among them, the seed vigor-related QTLs on 8 chromosomes (1B, 3A, 3B, 4D 5A, 5B, 6D, and 7A) (Supplementary Table S5) have not previously been reported, indicating that these may be new related QTLs.

### Prediction of candidate genes related to seed vigor in wheat

Seed germination is a complex physiological process involving the metabolism of carbohydrates, lipids, proteins, and other substances. In this study, 56 genes were found in the marker interval AX-94643729 ~ AX-110529646 (physical range 707,412,449–710,959,479 bp) on chromosome 5B, and 7 candidate genes related to seed vigor were screened out, according to gene function annotation and the results of GO and KEGG analyses. The functional annotation of the candidate gene *TraesCS5B01G564900* is an invertase/pectin methylesterase (PME) inhibitor family protein. Hothorn et al.^31^ demonstrated that PME and invertase are key enzymes for plant carbohydrate metabolism, which are involved in the development of plant roots, stems, and fruits. Inhibitors of these two enzymes form the sequence family of extracellular proteins and participate in the regulation of carbohydrate metabolism. The functional annotation of the candidate gene *TraesCS5B01G562600* and *TraesCS5B01G562700* are GDSL esterase/lipase protein. The GDSL lipase gene has been shown to be involved in plant growth and development, lipid metabolism, and stress responses^32^. *BnLIP1*, the GDSL lipase gene from *Brassica napus*, changes with seed germination and may also be involved in regulating other physiological processes^33^. During the germination of aging seeds, the participation of transcription factors can ensure that the target gene is expressed at a specific time in a specific space. The functional annotation of the candidate gene *Traescs5b01g564200* is a *Trihelix* transcription factor. Studies have shown that the *Trihelix* transcription factor is involved in plant embryogenesis and seed development ^24,34^, along with the plant abiotic stress response^35^.

Cytokinins are a class of plant hormones that promote cell division, bud formation, and growth and are related to seed development^36,37^. Jameson et al.^37^ detected cytokinins in bloated pea seeds and found that they were actively synthesized during germination. In this study, the candidate genes *TRAESCS5B01G561300.1* and *TRAESCS5B01G561400.1* were found to be functionally related to cytokinin nucleoside 5-monophosphate phosphatase. Other factors involved in plant growth and development are members of the *WD40* protein family, which act as scaffolds in biomacromolecule interactions and exist widely in eukaryotes. In *Arabidopsis*, these proteins are considered to be the key regulatory factors in signal transmission during development and stress. For example, the *WD40* repeat sequence protein *AGB1* in *Arabidopsis* negatively regulates the signal transduction of auxin and affects cell division in fruits, hypocotyls, and roots^38,39^. *GIGANTUS1 (GTS1*), a member of the *WD40* protein superfamily, is highly expressed in *Arabidopsis* embryos and controls seed germination, growth, and biomass accumulation through interactions with ribosomal protein chaperones^40^. Nucleosome remodeling factor complex components 101 and 102, which contain *WD40* repeat sequences in maize, regulate seed germination, plant height, flowering time, etc. by regulating chromatin modification^41^. The functional annotation of the candidate gene *Traescs5b01g562100.1* was that of a *WD40* repeat domain and the gene was also found to be closely related to seed vigor. In future studies, the expression of these seven genes during the germination of aging seeds should be analyzed to verify their functions using transgene experiments, following by investigations into the mechanisms underlying their functions.

## Materials and methods

### Test material

A wheat DH population, derived from a Hanxuan 10 × Lumai 14 cross was used as experimental material. The DH population with 150 lines was established by in vitro culture of anthers. The female parent, Hanxuan 10 is a dryland variety, bred by the Shanxi Academy of Agricultural Sciences in 1966; it exhibits excellent resistance to drought, barren soil, and cold temperatures. The male parent, Lumai 14, is a high-yield variety grown on irrigated land, which was bred in 1986 at the Yantai Agricultural Science Research Institute. The population was planted at Shanxi Agricultural University (37° 25′ N, 112° 25′ E) in 2017–2018. The field design consisted of randomized complete blocks with three replications. Each plot consisted of two rows of 2 m long, with 25 cm between rows. Forty seeds were sown in each row. Water and fertilizer management during the growth period was the same as that used in the local production practice. The seeds were harvested in June 2018.

### Experiment methods

The experiment was carried out at Shanxi Agricultural University between January and July, 2019. First, the seeds were artificially aged. There were six controlled-environment treatments for each line, 100 seeds for each treatment, and three replicates. Seeds were placed in an artificial climate incubator at a temperature of 48 °C and relative humidity of 100%. The seeds were aged under dark conditions for 0 h (a), 24 h (b), 36 h (c), 48 h (d), 60 h (e), and 72 h (f), respectively. The aged seeds were dried at room temperature for 3 days and then the standard germination test was carried out. The seeds were first disinfected, soaked in a 0.1% HgCl_2_ solution for 15 min, and washed three times with distilled water. The seeds were placed with the ventral groove facing down evenly in a culture dish (12 cm in diameter) with two layers of filter paper. An appropriate amount of distilled water was added and the dish was put in an artificial climate incubator (25 °C, 75% relative humidity) for germination. During germination, the photoperiod was 16 h light / 8 h dark. The plates were checked regularly and distilled water was replenished with an appropriate amount every day. From the third day of culture, the germination number for each line was counted. The shortest protruding part of seed radicle was the same as the length of seed, which was regarded as normal germination. The seedling length (SL), root length (RL), and seedling weight (SW) of each line were measured on the seventh day of culture.

Calculation of wheat seed vigor related traits:

$$\begin{aligned} & {\text{Germination}}\;{\text{percentage}}\;({\text{GP}}) = \, \left( {{\text{n}}_{{7}} /{ 1}00} \right) \, \times { 1}00\% \\ & {\text{Germination}}\;{\text{energy}}\left( {{\text{GE}}} \right) = \, \left( {{\text{n}}_{{3}} /{ 1}00} \right) \, \times { 1}00\% \\ & {\text{Germination}}\;{\text{index }}\left( {{\text{GI}}} \right) \, = \, \Sigma {\text{Gt }}/{\text{ Dt}} \\ & {\text{Vigor}}\;{\text{index }}\left( {{\text{VI}}} \right) \, = {\text{GI }} \times {\text{ SL}} \\ & {\text{Simple}}\;{\text{ vigor }}\;{\text{index }}\left( {{\text{SVI}}} \right) \, = {\text{ GP }} \times {\text{ SL}} \\ \end{aligned}$$

In the formula above, n_3_ is the number of germinated seeds on the 3rd day of culture, n_7_ is the number of germinated seeds on the 7th day of culture, Gt is the daily germination number, Dt is the corresponding germination days of Gt.

### Data analysis

Relevant t-test and analysis of variance (ANOVA) were carried out by the statistical software package SPSS 16.0, frequency distribution map was carried out by Excel 2007, phenotypic correlation analysis map and phylogenetic clustering map were created in the R-package corrplot (version 0.84)^42^ and clusterProfiler (version 3.16)^43^, respectively. The heritability of the traits was analyzed by analysis of variance. The formula of heritability is: *H*_*B*_^2^ = *σ*_*g*_^2^/(*σ*_*g*_^2^ + *σ*_*e*_^2^/*r*) × 100%, in which *σ*_*g*_^2^ is the genetic variance, *σ*_*e*_^2^ is the random error variance, and *r* is the number of trials repeated.

### QTL mapping

The genetic map of the DH population was constructed by Jing Ruilian’s team at the Institute of Crop Science, Chinese Academy of Agricultural Sciences. The map was created by integrating the same genetic position with SNP markers and SSR markers. The final number of markers was 1854, of which there were 1630 660 K SNP markers. There were 224 SSR markers, the total length was 4082.44 cM, and the average distance between the markers was 2.20 cM, including 30 linkage groups.

In recent years, the statistical methods commonly used in QTL mapping are mainly MCIM, ICIM and GCIM^44^. In this study, QTL analysis was analyzed by IciMapping software for seed vigor-related traits after artificial aging treatment, with inclusive composite interval mapping (ICIM)^45^. The average value of the three repeated measurements of seed vigor-related traits in the DH population was used as the phenotypic value. The walking speed for all QTL detections was chosen at 1.0 centimorgans (cM), with *P* = 0.001 in stepwise regression. Based on 2000 permutations at a probability of 0.01, the significant QTL threshold was obtained. Combined with this genetic map, a main-effect and epistatic QTL mapping were performed. At the same time, QTL IciMapping software was used to map the distribution of QTL loci on wheat chromosomes. The QTLs are named after “Q + trait name + processing time + chromosome”. The GCIM method in the software QTL.gCIMapping from the R website (https://cran.r-project.org/web/packages/QTL.gCIMapping/index.html) was also used to identify QTLs for the above traits, with the purpose of identifying the results from the ICIM method; the critical LOD scores for a significant QTL was also set at 2.5, and the walking speed for the genome-wide scan was set at 1 cM.

### The GO and KEGG analyses

The online prediction website (https://geneontology.org/) was used to perform GO (Gene Ontology) enrichment analysis on candidate genes. KEGG (Kyoto Encyclopedia of Genes and Genomes) analysis was performed on candidate genes using the KEGG analysis website (https://www.kegg.jp/keg/kegg1.html))^46,47^.

## Conclusion

A total of 49 additive QTLs of wheat seed vigor-related traits were detected and distributed on 12 chromosomes including 1B, 2D, 3A, 3B, 3D, 4A, 4D, 5A, 5B, 5D, 6D, and 7A. The chromosomes with more QTL loci were 5D, 5B, and 5A. Three hotspots of additive QTLs were detected on chromosomes 5A, 5B, and 5D. Seven genes related to seed vigor traits were screened from the hot spots on chromosome 5B, which were *TRAESCS5B01G564900, TRAESCS5B01G564200, TRAESCS5B01G562600, TraesCS5B02G562700, TRAESCS5B01G561300, TRAESCS5B01G561400*, and *TRAESCS5B01G562100.* These genes may be involved in the regulation of several processes including carbohydrate and lipid metabolism, transcription, and cell division, during the germination of aging seeds. These newly detected QTLs, SNP markers, and related candidate genes provide valuable information for molecular marker-assisted selective breeding of high-vigor wheat seeds.

## Supplementary information

Supplementary Information 1.

Supplementary Information 2.

## Data Availability

The experimental materials and relevant data involved in this paper can be used publicly.

## References

[1] Chen LT (2016). Seed vigor evaluation based on adversity resistance index of wheat seed germination under stress conditions. Chin. J. Appl. Ecol..

[2] Han Z (2014). QTLs for seed vigor-related traits identified in maize seeds germinated under artificial aging conditions. PLoS ONE.

[3] Cao LY, Zhu J, Ren LF, Zhao ST, Yan QC (2002). Mapping QTLs and epistasis for seedling vigor in rice (*Oryza sativa* L). Acta Agron. Sin..

[4] Brits GJ, Brown NAC, Calitz FJ, Van Staden J (2015). Effects of storage under low temperature, room temperature and in the soil on viability and vigour of *Leucospermum cordifolium*(Proteaceae)seeds. S. Afr. J. Bot..

[5] Ventura L (2012). Understanding the molecular pathways associated with seed vigor. Plant Physiol. Biochem..

[6] Wang XF, Cong ZJ (1997). The biological basis of seed vigor and the research progress of improving and maintaining seed vigor. Seed.

[7] Zheng YL (2017). Comparative study on determination methods of wheat seed vigor. J. Jiangsu Agric. Sci..

[8] Sun Q, Wang JH, Sun BQ (2007). Advances on seed vigor physiological and genetic mechanisms. Sci. Agric. Sin..

[9] Gao HY, Jing LQ, Chen L, Ju J, Wang YX, Zhu JG (2016). Effects of elevated atmospheric CO_2_ and temperature on seed vigor of rice under open-air field conditions. Chin. J. Rice Sci..

[10] Catusse J, Job C, Job D (2008). Transcriptome-and proteome-wide analyses of seed germination. Comptes Rendus Biol..

[11] Li T (2017). Regulation of seed vigor by manipulation of raffinose family oligosaccharides (RFOs) in maize and *Arabidopsis*. Mol. Plant..

[12] Cheng XY (2013). A rice lectin receptor-like kinase that is involved in innate immune responses also contributes to seed germination. Plant J..

[13] Kaur H (2015). Differentially expressed seed aging responsive heat shock protein OsHSP18.2 implicates in seed vigor, longevity and improves germination and seedling establishment under biotic stress. Front. Plant Sci..

[14] Chatelain E (2013). Evidence for participation of the methionine sulfoxide reductase repair system in plant seed longevity. Proc. Natl. Acad. Sci. USA.

[15] Wei YD (2015). Protein repair L-isoaspartylmethyltransferase1 (PIMT1) in rice improves seed longevity by preserving embryo vigor and viability. Plant Mol. Biol..

[16] Petla BP (2016). Rice PROTEIN l-ISOASPARTYL METHYLTRANSFERASE isoforms differentially accumulate during seed maturation to restrict deleterious isoAsp and reactive oxygen species accumulation and are implicated in seed vigor and longevity. New Phytol..

[17] Sun YL, Liu HM, Xu QG (2017). Effects of cadmium stress on rice seed germination characteristics. Chin. J. Rice Sci..

[18] Zhang AP, Qian Q, Gao ZY (2018). Research advances on rice seed vigor. Chin. J. Rice Sci..

[19] Miura K, Lin Y, Yano M, Nagamine T (2002). Mapping quantitative trait loci controlling seed longevity in rice (*Oryza sativa* L.). Theor. Appl. Genet..

[20] Clerkx E, Vries H, Ruys G, Groot S, Koornneef M (2004). Genetic differences in seed longevity of various *Arabidopsis* mutants. Physiol. Plant..

[21] Nagel M (2011). Seed longevity in oilseed rape (*Brassica napus* L.)—genetic variation and QTL mapping. Plant Genet. Resour. C.

[22] Nagel M (2016). Barley seed aging: Genetics behind the dry elevated pressure of oxygen aging and moist controlled deterioration. Front. Plant Sci..

[23] Arif MAR, Nagel M, Lohwasser U, Börner A (2017). Genetic architecture of seed longevity in bread wheat (*Triticum aestivum* L.). J. Biosci. (Bangalore).

[24] Xie LX (2014). Identification and fine mapping of quantitative trait loci for seed vigor in germination and seedling establishment in rice. J. Integr. Plant Biol..

[25] Li CS (2017). QTL identification and fine mapping for seed storability in rice (*Oryza sativa* L.). Euphytica.

[26] Abe A (2012). *OsGA20ox1*, a candidate gene for a major QTL controlling seedling vigor in rice. Theor. Appl. Genet..

[27] Ye SQ (2017). Effects of high temperature on germination rate of rice seed. Chi. Rice..

[28] Shi Y (2016). Genetic dissection of seed vigour traits in maize (*Zea mays* L.) under low-temperature conditions. J. Genet..

[29] Misheva S, Lohwasser U, Börner A (2010). Genetic mapping within the wheat D genome reveals QTL for germination, seed vigour and longevity, and early seedling growth. Euphytica.

[30] Zuo JH (2018). Genome-wide linkage mapping reveals QTLs for seed vigor-related traits under artificial aging in common wheat (*Triticum aestivum*). Front. Plant Sci..

[31] Hothorn M, Wolf S, Aloy P, Greiner S, Scheffzek K (2005). Structural insights into the target specificity of plant invertase and pectin methylesterase inhibitory proteins. Plant Cell..

[32] Yu HF (2018). Sequence analysis of GDSL esterase/lipase genes in *Helianthus annuus* L. J. China Agric. Univ..

[33] Ling H (2006). Isolation and characterization of a homologous to lipase gene from *Brassica napus*. Russ. J. Plant Physiol..

[34] Tzafrir I (2004). Identification of genes required for embryo development in *Arabidopsis*. Plant Physiol..

[35] Yu C (2018). A Trihelix family gene, mediates cold and drought tolerance by interacting with *SnRK1* in tomato. Plant Sci..

[36] Jameson P, Song J (2015). Cytokinin: a key driver of seed yield. J. Exp. Bot..

[37] Jameson P, Dhandapani P, Novak O, Song J (2016). Cytokinins and expression of SWEET, SUT, CWINV and AAP genes increase as pea seeds germinate. Int. J. Mol. Sci..

[38] Mudgil Y (2016). Photosynthate regulation of the root system architecture mediated by the heterotrimeric G protein complex in arabidopsis. Front. Plant Sci..

[39] Xu P (2019). Phytochrome B and AGB1 coordinately regulate photomorphogenesis by antagonistically modulating PIF3 stability in arabidopsis. Mol. Plant..

[40] Gachomo EW, Jimenez-Lopez JC, Baptiste LJ, Kotchoni SO (2014). GIGANTUS1 (GTS1), a member of Transducin/WD40 protein superfamily, controls seed germination, growth and biomass accumulation through ribosome-biogenesis protein interactions in *Arabidopsis thaliana*. BMC Plant Biol..

[41] Mishra A, Puranik S, Bahadur R, Prasad M (2012). The DNA-binding activity of an AP2 protein is involved in transcriptional regulation of a stress-responsive gene, Si WD40, in foxtail millet. Genomics.

[42] Wei TY, Simko V (2013). corrplot: visualization of a correlation matrix. MMWR Morbid. Mortal. Week..

[43] Yu G, Wang LG, Han Y, He QY (2012). Clusterprofiler: an r package for comparing biological themes among gene clusters. Omics.

[44] Wen YJ (2019). An efficient multi-locus mixed model framework for the detection of small and linked QTLs in F_2_. Briefings Bioinform..

[45] Li HH, Ye GY, Wang JK (2007). A modified algorithm for the improvement of composite interval mapping. Genetics.

[46] Kanehisa M, Goto S, Kawashima S, Okuno Y, Hattori M (2004). The KEGG resource for deciphering the genome. Nucleic Acids Res..

[47] Kanehisa M, Goto S (2000). KEGG: kyoto encyclopedia of genes and genomes. Nucleic Acids Res..

