# Supplementary material for: QTL mapping and candidate gene analysis of seed vigor-related traits during artificial aging in wheat (Triticum aestivum)
Source: Sci Rep. 2020 Dec 16;10:22060. doi: 10.1038/s41598-020-75778-z (PMC7745025; doi:10.1038/s41598-020-75778-z)
Supplement: Supplementary file 2 — Supplementary Information 2. [file 41598_2020_75778_MOESM2_ESM.docx]

| Supplementary Table S1 Phenotypic data analysis of seed vigor traits of wheat DH population and their parents under different aging treatments | | | | | | | | | | |
| --- | --- | --- | --- | --- | --- | --- | --- | --- | --- | --- |
| Traits | Hanxuan 10 | Lumai 14 | T - value | DH population | | | | | | |
|  |  |  |  | Minimum value | Maximum value | Average value | Variation  coefficients | Skewness | Kurtosis | Heritability（%） |
| GPa | 0.92±0.02 | 0.93±0.02 | 0.16 | 0.35 | 1.00 | 0.85 | 0.17 | -1.03 | 0.61 | 90 |
| GPb | 0.80±0.01 | 0.70±0.02 | 3.58^**^ | 0.10 | 1.00 | 0.69 | 0.34 | -0.17 | -1.27 | 95 |
| GPc | 0.67±0.03 | 0.58±0.04 | 5.60^**^ | 0.06 | 1.00 | 0.58 | 0.48 | 0.08 | -1.42 | 97 |
| GPd | 0.54±0.02 | 0.47±0.01 | 1.99 | 0.02 | 0.98 | 0.48 | 0.64 | 0.30 | -1.33 | 97 |
| GPe | 0.43±0.03 | 0.36±0.02 | 2.54^*^ | 0.00 | 0.99 | 0.38 | 0.82 | 0.67 | -0.95 | 98 |
| GPf | 0.40±0.01 | 0.21±0.01 | 4.18^**^ | 0.00 | 0.97 | 0.27 | 1.11 | 1.13 | -0.12 | 98 |
| GEa | 0.86±0.04 | 0.86±0.02 | 0.28 | 0.25 | 1.00 | 0.76 | 0.27 | -0.41 | -1.11 | 93 |
| GEb | 0.70±0.02 | 0.62±0.03 | 3.21^*^ | 0.08 | 1.00 | 0.61 | 0.44 | 0.10 | -1.52 | 96 |
| GEc | 0.60±0.01 | 0.52±0.02 | 7.07^**^ | 0.04 | 0.99 | 0.50 | 0.59 | 0.30 | -1.41 | 97 |
| GEd | 0.49±0.02 | 0.43±0.01 | 2.00 | 0.00 | 0.96 | 0.38 | 0.80 | 0.65 | -0.97 | 98 |
| GEe | 0.33±0.01 | 0.27±0.02 | 2.89^*^ | 0.00 | 0.97 | 0.28 | 1.11 | 1.13 | -0.16 | 98 |
| GEf | 0.10±0.01 | 0.10±0.01 | 0.24 | 0.00 | 0.96 | 0.18 | 1.58 | 1.75 | 1.69 | 98 |
| GIa | 19.52±0.48 | 19.42±0.38 | 0.26 | 7.56 | 22.22 | 17.75 | 0.22 | -0.53 | -0.81 | 93 |
| GIb | 16.62±0.07 | 14.55±0.26 | 3.88^**^ | 1.78 | 22.15 | 14.29 | 0.39 | 0.02 | -1.44 | 97 |
| GIc | 13.87±0.07 | 12.10±0.39 | 7.76^**^ | 1.04 | 22.05 | 11.86 | 0.54 | 0.22 | -1.43 | 98 |
| GId | 11.07±0.46 | 9.72±0.89 | 1.87 | 0.07 | 21.48 | 9.34 | 0.72 | 0.50 | -1.13 | 98 |
| GIe | 7.73±1.00 | 6.79±1.37 | 2.16 | 0.00 | 21.46 | 7.00 | 0.95 | 0.95 | -0.47 | 99 |
| GIf | 5.72±0.99 | 3.22±0.74 | 4.18^**^ | 0.00 | 20.99 | 4.75 | 1.30 | 1.50 | 0.96 | 99 |
| SLa | 13.46±0.24 | 11.43±0.79 | 6.23^**^ | 9.01 | 17.48 | 12.33 | 0.14 | 0.33 | -0.09 | 91 |
| SLb | 12.02±0.21 | 10.71±0.49 | 4.22^**^ | 5.67 | 15.07 | 11.27 | 0.15 | -0.29 | -0.03 | 88 |
| SLc | 10.52±0.33 | 9.63±0.26 | 3.41^**^ | 5.82 | 14.40 | 9.99 | 0.19 | -0.06 | -0.56 | 88 |
| SLd | 9.21±0.03 | 8.35±0.15 | 5.56^**^ | 0.19 | 14.34 | 8.26 | 0.32 | -0.51 | 0.94 | 86 |
| SLe | 7.36±0.20 | 5.97±0.43 | 1.86 | 0.00 | 14.55 | 6.69 | 0.47 | -0.01 | -0.18 | 93 |
| SLf | 5.34±0.18 | 3.72±0.16 | 1.98 | 0.00 | 12.93 | 4.65 | 0.74 | 0.54 | -0.46 | 92 |
| RLa | 9.55±0.93 | 8.75±1.22 | 6.29^**^ | 3.75 | 16.21 | 8.36 | 0.32 | 0.40 | -0.68 | 93 |
| RLb | 8.19±0.85 | 8.30±0.84 | 0.30 | 3.42 | 14.47 | 7.57 | 0.36 | 0.32 | -0.81 | 91 |
| RLc | 7.53±1.64 | 7.50±1.33 | 0.10 | 2.70 | 13.61 | 6.82 | 0.41 | 0.24 | -1.11 | 91 |
| RLd | 6.37±0.49 | 6.97±0.53 | 1.76 | 0.08 | 13.68 | 5.78 | 0.49 | 0.20 | -0.83 | 90 |
| RLe | 5.34±0.74 | 5.59±0.84 | 0.44 | 0.00 | 13.33 | 4.78 | 0.61 | 0.44 | -0.35 | 92 |
| RLf | 3.99±0.76 | 3.66±0.62 | 0.40 | 0.00 | 13.73 | 3.42 | 0.83 | 0.95 | 0.68 | 92 |
| SWa | 0.41±0.02 | 0.33±0.02 | 4.76^**^ | 0.23 | 0.53 | 0.35 | 0.14 | 0.36 | 1.13 | 81 |
| SWb | 0.37±0.01 | 0.31±0.01 | 7.35^**^ | 0.20 | 0.49 | 0.33 | 0.15 | 0.22 | 0.41 | 86 |
| SWc | 0.32±0.05 | 0.30±0.03 | 3.47^**^ | 0.18 | 0.44 | 0.31 | 0.17 | 0.09 | -0.32 | 81 |
| SWd | 0.30±0.01 | 0.26±0.01 | 4.10^**^ | 0.05 | 0.40 | 0.27 | 0.26 | -0.72 | 0.78 | 83 |
| SWe | 0.26±0.01 | 0.23±0.01 | 1.76 | 0.00 | 0.39 | 0.23 | 0.38 | -0.88 | 0.48 | 88 |
| SWf | 0.23±0.01 | 0.18±0.01 | 3.00^*^ | 0.00 | 0.43 | 0.18 | 0.57 | -0.15 | -0.54 | 87 |
| VIa | 260.46±21.85 | 222.58±15.57 | 6.05^**^ | 75.62 | 334.70 | 219.58 | 0.26 | -0.22 | -0.88 | 93 |
| VIb | 201.46±6.50 | 161.87±11.77 | 4.85^**^ | 19.43 | 293.01 | 162.58 | 0.43 | 0.13 | -1.22 | 96 |
| VIc | 151.10±11.26 | 125.42±8.15 | 6.36^**^ | 7.23 | 283.00 | 123.79 | 0.62 | 0.42 | -1.02 | 97 |
| VId | 108.58±9.08 | 91.04±8.93 | 2.55^*^ | 0.03 | 264.40 | 88.59 | 0.83 | 0.79 | -0.50 | 97 |
| VIe | 58.85±5.34 | 54.78±6.75 | 0.83 | 0.00 | 248.54 | 61.59 | 1.15 | 1.25 | 0.33 | 98 |
| VIf | 34.74±5.86 | 22.27±4.25 | 4.71^**^ | 0.00 | 235.68 | 37.79 | 1.60 | 1.88 | 2.33 | 98 |
| SVIa | 12.35±1.06 | 10.58±0.76 | 5.99^**^ | 3.60 | 15.18 | 10.56 | 0.22 | -0.32 | -0.58 | 91 |
| SVIb | 9.76±0.30 | 7.76±0.52 | 4.30^**^ | 1.09 | 13.25 | 7.89 | 0.38 | -0.03 | -1.12 | 95 |
| SVIc | 7.30±0.54 | 5.95±0.39 | 5.19^**^ | 0.42 | 12.85 | 6.05 | 0.56 | 0.30 | -1.07 | 97 |
| SVId | 5.32±0.41 | 4.38±0.32 | 2.69^*^ | 0.01 | 12.29 | 4.46 | 0.76 | 0.60 | -0.79 | 97 |
| SVIe | 3.24±0.69 | 2.85±0.73 | 1.80 | 0.00 | 11.47 | 3.20 | 1.02 | 1.05 | -0.13 | 98 |
| SVIf | 2.40±0.42 | 1.47±0.31 | 4.30^**^ | 0.00 | 10.90 | 2.01 | 1.42 | 1.68 | 1.77 | 98 |

Note: GP, GE, GI, SL, RL, SW, VI, SVI=Germination Percentage, Germinating Energy, Germination Index, Seedling Lengh, Root Length, Seedling Weight,Vigor Index,and Simple Vigor Index, respectively. a, b, c, d, e, and f represent aging for 0 h, aging 24 h, aging 36 h, aging 48 h, aging 60 h and aging 72 h, respectively. * and ** represent the significance level at P<0.05 and P<0.01, respectively.

Supplementary Table S2 Additive QTLs for seed vigor traits detected by the GCIM method in wheat DH lines

| Trait | QTL | Chr. | Position (cM) | LOD score | Left_marker | Right_marker | PVE  (%) | Add |
| --- | --- | --- | --- | --- | --- | --- | --- | --- |
| GEf | *QGEf5A* | 5A | 16.493 | 3.4185 | AX-94524640 | AX-89452330 | 10.49 | -0.0901 |
| GIe | *QGIe5A* | 5A | 40.154 | 2.6289 | AX-89381362 | AX-89381362 | 8.19 | 1.9115 |
| SLb | *QSLb5A* | 5A | 69.183 | 6.0518 | Xgwm415 | Xgwm415 | 15.76 | 0.6729 |
| SLc | *QSLc5A* | 5A | 69.183 | 2.6339 | Xgwm415 | Xgwm415 | 5.93 | 0.5112 |
| SLe | *QSLe5A* | 5A | 69.183 | 5.2468 | Xgwm415 | Xgwm415 | 13.98 | 1.1775 |
| VIf | *QVIf5A* | 5A | 18.382 | 2.8656 | AX-94524640 | AX-89452330 | 5.27 | -16.587 |
| GIf | *QGIf5B* | 5B | 0 | 2.9591 | AX-110653882 | AX-110653882 | 7.03 | -1.7409 |
| SLa | *QSLa5B* | 5B | 191.024 | 2.8643 | AX-109384574 | AX-109384574 | 7.63 | 0.4774 |
| SLb | *QSLb5B* | 5B | 188.638 | 2.9973 | AX-94700213 | AX-94700213 | 7.33 | 0.459 |
| SLd | *QSLd5B* | 5B | 229.622 | 4.1956 | AX-110529646 | AX-110529646 | 9.44 | 0.8281 |
| SLe | *QSLe5B* | 5B | 229.622 | 2.5219 | AX-110529646 | AX-110529646 | 6.31 | 0.7911 |
| SLf | *QSLf5B* | 5B | 229.622 | 3.7495 | AX-110529646 | AX-110529646 | 9.61 | 1.0737 |
| RLc | *QRLc5B* | 5B | 229.622 | 2.7902 | AX-110529646 | AX-110529646 | 7.44 | 0.7566 |
| RLe | *QRLe5B* | 5B | 229.622 | 3.8566 | AX-110529646 | AX-110529646 | 7.19 | 0.8881 |
| RLf | *QRLf5B* | 5B | 229.622 | 3.3787 | AX-110529646 | AX-110529646 | 8.84 | 0.8486 |
| RLd | *QRLd5B* | 5B | 229.622 | 2.9827 | AX-110529646 | AX-110529646 | 7.65 | 0.7787 |
| SLf | *QSLf5D* | 5D | 18.912 | 4.6584 | Xgdm3 | Xgdm3 | 12.17 | 1.2083 |
| VIe | *QVIe5D* | 5D | 18.912 | 4.7702 | Xgdm3 | Xgdm3 | 13.23 | 25.7111 |
| SVIf | *QSVIf5D* | 5D | 12.895 | 4.5496 | AX-89752452 | AX-89752452 | 11.93 | 0.9907 |

Note: GE, GI, SL, RL, VI, and SVI = Germinating energy, Germination index, Seedling lengh, Root length, Vigor index, and Simple vigor index, respectively. PVE = percentage of phenotypic variance explained by each QTL, Add = additive effect. a, b, c, d, e, and f represent aging for 0 h, aging 24 h, 36 h, 48 h, 60 h, and 72 h, respectively.

Supplementary Table S3 TOP7 GO enrichment analysis of the candidate genes

| GO ID | GO Term | number_of Genes | Genes |
| --- | --- | --- | --- |
| GO:0005488 | binding | 22 | *TraesCS5B02G561000,TraesCS5B02G561100,TraesCS5B02G561500,TraesCS5B02G562100,TraesCS5B02G562200,TraesCS5B02G562800,TraesCS5B02G563300,TraesCS5B02G563400,TraesCS5B02G563600,TraesCS5B02G563800,TraesCS5B02G563900,TraesCS5B02G564200,TraesCS5B02G565000,TraesCS5B02G565100,TraesCS5B02G565300,TraesCS5B02G565400,TraesCS5B02G565500,TraesCS5B02G565900,TraesCS5B02G566100,TraesCS5B02G566200,TraesCS5B02G566700,TraesCS5B02G566900* |
| GO:0008152 | metabolic process | 13 | *TraesCS5B02G561300,TraesCS5B02G561400,TraesCS5B02G562200,TraesCS5B02G563700,TraesCS5B02G563800,TraesCS5B02G564900,TraesCS5B02G565000,TraesCS5B02G565100,TraesCS5B02G565300,TraesCS5B02G565400,TraesCS5B02G565900,TraesCS5B02G566000,TraesCS5B02G566700* |
| GO:0003824 | catalytic activity | 12 | *TraesCS5B02G561300,TraesCS5B02G561400,TraesCS5B02G562600,TraesCS5B02G562700,TraesCS5B02G565000,TraesCS5B02G565100,TraesCS5B02G565800,TraesCS5B02G565900,TraesCS5B02G566000,TraesCS5B02G566100,TraesCS5B02G566200,TraesCS5B02G566700* |
| GO:0009987 | cellular process | 11 | *TraesCS5B02G561300,TraesCS5B02G561400,TraesCS5B02G562200,TraesCS5B02G562500,TraesCS5B02G563700,TraesCS5B02G563800,TraesCS5B02G563900,TraesCS5B02G565000,TraesCS5B02G565100,TraesCS5B02G565300,TraesCS5B02G565400* |
| GO:0016020 | membrane | 9 | *TraesCS5B02G561600,TraesCS5B02G562100,TraesCS5B02G562500,TraesCS5B02G564000,TraesCS5B02G564300,TraesCS5B02G564400,TraesCS5B02G564700,TraesCS5B02G566400,TraesCS5B02G566500* |
| GO:0065007 | biological regulation | 8 | *TraesCS5B02G561300,TraesCS5B02G561400,TraesCS5B02G562200,TraesCS5B02G563700,TraesCS5B02G563800,TraesCS5B02G564900,TraesCS5B02G565300,TraesCS5B02G565400* |
| GO:0044425 | membrane part | 8 | *TraesCS5B02G561600,TraesCS5B02G562100,TraesCS5B02G564000,TraesCS5B02G564300,TraesCS5B02G564400,TraesCS5B02G564700,TraesCS5B02G566400,TraesCS5B02G566500* |

Supplementary Table S4 KEGG pathway enrichment analysis of the candidate genes

| ID | Term | Genes |
| --- | --- | --- |
| K13457 | disease resistance protein RPM1-like | *TraesCS5B02G562800，TraesCS5B02G563600，TraesCS5B02G565500，TraesCS5B02G566900* |
| K01083 | 3-phytase | *TraesCS5B02G562600，TraesCS5B02G562700* |
| K01113 | alkaline phosphatase D | *TraesCS5B02G562600，TraesCS5B02G562700* |
| K14209 | solute carrier family 36 (proton-coupled amino acid transporter) | *TraesCS5B02G562600，TraesCS5B02G562700* |
| K21026 | cetylajmaline esterase | *TraesCS5B02G562600，TraesCS5B02G562700* |
| K21831 | cholinesterase | *TraesCS5B02G562600，TraesCS5B02G562700* |
| K08266 | target of rapamycin complex subunit LST8 | *TraesCS5B02G562100* |
| K12662 | U4/U6 small nuclear ribonucleoprotein PRP4 | *TraesCS5B02G562100* |
| K14554 | U3 small nucleolar RNA-associated protein 21 | *TraesCS5B02G562100* |
| K14556 | U3 small nucleolar RNA-associated protein 12 | *TraesCS5B02G562100* |
| K14855 | ribosome assembly protein 4 | *TraesCS5B02G562100* |
| K14963 | COMPASS component SWD3 | *TraesCS5B02G562100* |
| K18643 | katanin p80 WD40 repeat-containing subunit B1 | *TraesCS5B02G562100* |
| K22193 | G protein-coupled receptor 89 | *TraesCS5B02G562100* |
| K04435 | C-Jun-amino-terminal kinase-interacting p | *TraesCS5B02G564200* |
| K13171 | serine and arginine repetitive matrix 1 | *TraesCS5B02G564200* |
| K17757 | ATP-dependent (S)-NAD(P)H-hydrate dehydra | *TraesCS5B02G564200* |
| K22522 | cytokinin riboside 5'-monophosphate phosphoribohydrolase | *TraesCS5B02G561300,TraesCS5B02G561400* |

Supplementary Table S5 QTLs on the same chromosome found by our study and previous studies

| Traits | QTL | Position(cM) | Marker interval/Nearest marker | Origin |
| --- | --- | --- | --- | --- |
| MGT | *QaMGT.cas-2DS.2* | 51 | *IWB12962~IWB11197* | Zuo et al[29] |
| MGT | *QaMGT.cas-2DS.2* | 46 | *IWB21991~IWB75065* | Zuo et al |
| MGR | *QaMGR.cas-2DS.2* | 48 | *IWB21991~IWB75065* | Zuo et al |
| MGR | *QaMGR.cas-2DS.2* | 51 | *IWB12962~IWB11197* | Zuo et al |
| MGR | *QaMGR.cas-2DS.2* | 47 | *IWB21991~IWB75065* | Zuo et al |
| GI | *QaGI.cas-2DL* | 97 | *IWB43924~IWB28458* | Zuo et al |
| GR | *QaGR.cas-2DL* | 97 | *IWB43924~IWB28458* | Zuo et al |
| Z | *QaZ.cas-2DS.2* | 48 | *IWB21991~IWB75065* | Zuo et al |
| FCGR | *QaFCGR.cas-2DS.2* | 51 | *IWB12962~IWB11197* | Zuo et al |
| RL | *QRLd2D* | *162* | *AX-108820505~ AX-109351504* | our study |
| CVt | *QCVt.ipk-2D* | - | *Xgwm261* | Misheva et al[28] |
| GI | *QaGI.cas-3DL* | *57* | *IWB34976~IWB25194* | Zuo et al |
| GR | *QaGR.cas-3DL* | *91* | *IWB52937~IWB17930* | Zuo et al |
| FCGR | *QaFCGR.cas-3DL* | *56* | *IWA5030~IWB34976* | Zuo et al |
| SWa | *QSWa3D* | *25* | *AX-94550177~ AX-111713694* | our study |
| GI | *QaGI.cas-4AL.1* | *60* | *IWB27577~IWA4079* | Zuo et al |
| GI | *QaGI.cas-4AL.2* | *79* | *IWB830~IWB1522* | Zuo et al |
| GI | *QaGI.cas-4AL.3* | *139* | *IWB21713~IWB28717* | Zuo et al |
| GI | *QaGI.cas-4AL.3* | *128* | *IWB23723~IWB49186* | Zuo et al |
| GR | *QaGR.cas-4AL.2* | *79* | *IWB830~IWB1522* | Zuo et al |
| GR | *QaGR.cas-4AL.3* | *139* | *IWB21713~IWB28717* | Zuo et al |
| Z | *QaZ.cas-4AS* | *43* | *IWB3572~IWB11606* | Zuo et al |
| Z | *QaZ.cas-4AL.2* | 71 | *IWB52955~IWB830* | Zuo et al |
| Z | *QaZ.cas-4AL.3* | 139 | *IWB21713~IWB28717* | Zuo et al |
| SWc | *QSWc4A* | 91 | *AX-94581303~ Xwmc468* | our study |
| SLa | *QSLa4D* | 16 | *AX-89703298~ AX-89421921* | our study |
| Clp | *Qclp.ipk-4D* | - | *Xgdm129* | Misheva et al |
| MGT | *QaMGT.cas-5DS* | 20 | *IWB60953~IWB44516* | Zuo et al |
| MGR | *QaMGR.cas-5DS* | 20 | *IWB60953~IWB44516* | Zuo et al |
| GI | *QaGI.cas-5DS* | 20 | *IWB60953~IWB44516* | Zuo et al |
| GPe | *QGPe5D* | 19 | *Xgdm3~ AX-109095227* | our study |
| GPf | *QGPe5D* | 19 | *Xgdm3~ AX-109095227* | our study |
| GEd | *QGPe5D* | 19 | *Xgdm3~ AX-109095227* | our study |
| GEe | *QGPe5D* | 19 | *Xgdm3~ AX-109095227* | our study |
| GId | *QGPe5D* | 19 | *Xgdm3~ AX-109095227* | our study |
| GIe | *QGPe5D* | 19 | *Xgdm3~ AX-109095227* | our study |
| GIf | *QGPe5D* | 19 | *Xgdm3~ AX-109095227* | our study |
| RLf | *QGPe5D* | 19 | *Xgdm3~ AX-109095227* | our study |
| VId | *QGPe5D* | 19 | *Xgdm3~ AX-109095227* | our study |
| VIe | *QGPe5D* | 19 | *Xgdm3~ AX-109095227* | our study |
| VIf | *QGPe5D* | 19 | *Xgdm3~ AX-109095227* | our study |
| SVId | *QGPe5D* | 19 | *Xgdm3~ AX-109095227* | our study |
| SVIe | *QGPe5D* | 19 | *Xgdm3~ AX-109095227* | our study |
| SVIf | *QGPe5D* | 19 | *Xgdm3~ AX-109095227* | our study |
| FinGP | *QLi-fingp.ipk-5D* | - | *Xgwm960,Xgdm3* | Misheva et al |
| SL | *Qslc.ipk-5D* | - | *Xgwm1122, Xgwm174* | Misheva et al |
| RSR | *QRsrc.ipk-5D* | - | *Xgwm1122* | Misheva et al |
| FCGP | *Qfcgp.ipk-5D* | - | *Xgwm1454* | Misheva et al |

Note: GP, GE, GI, SL, RL, SW, VI, SVI , MGT, MGR , Z, FCGR , CVt, Clp, FinGP, RSR, FCGP=Germination percentage, Germinating energy, Germination index, Seedling lengh, Root length, Seedling weight,Vigor index, Simple vigor index, Mean germination time, Mean germination rate, The synchrony index, First count germination ratio, Coefficient of variation of germination time, Cleoptile length, Final count germination percentage, Root seedling length ratio, and First count germination percentage, respectively. a, b, c, d, e, and f represent aging for 0 h, 24 h, 36 h, 48 h, 60 h and 72 h, respectively.
